# Inducible ASABF-Type Antimicrobial Peptide from the Sponge *Suberites domuncula*: Microbicidal and Hemolytic Activity *in Vitro* and Toxic Effect on Molluscs *in Vivo*†

**DOI:** 10.3390/md9101969

**Published:** 2011-10-19

**Authors:** Matthias Wiens, Heinz C. Schröder, Michael Korzhev, Xiao-Hong Wang, Renato Batel, Werner E. G. Müller

**Affiliations:** 1 ERC Advanced Grant Research Group at the Institute for Physiological Chemistry, University Medical Center of the Johannes Gutenberg University Mainz, Duesbergweg 6, Mainz D-55128, Germany; E-Mails: wiens@uni-mainz.de (M.W.); hschroed@uni-mainz.de (H.C.S.); korzhev@uni-mainz.de (M.K.); wxh0408@hotmail.com (X.-H.W.);; 2 Rudjer Boskovic Institute, Center for Marine Research, Giordano Paliaga 5, Rovinj HR-52210, Croatia; E-Mail: batel@cim.irb.hr

**Keywords:** sponges, *Suberites domuncula*, ASABF, antimicrobial peptides, apoptosis, *Bittium* sp

## Abstract

Since sponges, as typical filter-feeders, are exposed to a high load of attacking prokaryotic and eukaryotic organisms, they are armed with a wide arsenal of antimicrobial/cytostatic low-molecular-weight, non-proteinaceous bioactive compounds. Here we present the first sponge agent belonging to the group of ASABF-type antimicrobial peptides. The *ASABF* gene was identified and cloned from the demosponge *Suberites domuncula*. The mature peptide, with a length of 64 aa residues has a predicted pI of 9.24, and comprises the characteristic CSα β structural motif. Consequently, the *S. domuncula* ASABF shares high similarity with the nematode ASABFs; it is distantly related to the defensins. The recombinant peptide was found to display besides microbicidal activity, anti-fungal activity. In addition, the peptide lyses human erythrocytes. The expression of *ASABF* is upregulated after exposure to the apoptosis-inducing agent 2,2′-dipyridyl. During the process of apoptosis of surface tissue of *S. domuncula*, grazing gastropods (*Bittium* sp.) are attracted by quinolinic acid which is synthesized through the kynurenine pathway by the enzyme 3-hydroxyanthranilate 3,4-dioxygenase (HAD). Finally, the gastropods are repelled from the sponge tissue by the ASABF. It is shown that the effector peptide ASABF is sequentially expressed after the induction of the *HAD* gene and a *caspase*, as a central enzyme executing apoptosis.

## 1. Introduction

Sponges, as sessile filter feeders, are exposed to a huge number of microorganisms, including bacteria, fungi, and viruses, which are eliminated during the passage through the aquiferous sponge canal system [1]. It had been calculated that some sponge species filter up to 0.84 cm^3^ of water per second per cubic centimeter of sponge tissue through their canals to extract edible material [2]. In order to resist against those microorganisms that could be harmful for the sponge specimens, these animals have developed a wide array of bioactive compounds [3] that act highly specifically and efficiently. Among those are secondary metabolites interfering with distinct pathways of higher metazoans, such as tyrosine kinases (e.g., aeroplysinin), and replication of viruses, e.g., herpes simplex viruses (arabinofuranosyl adenine) or retroviruses (e.g., avarol) [4,5].

In addition to those directly acting inhibitory, secondary metabolites sponges have a very effective immune system [6]. Nevertheless it is well established that sponges are not provided with an antigen-specific adaptive immune system, even though they express proteins composed of two characteristic immunoglobulin (Ig)-like domains, e.g., in *Geodia cydonium* [7]. However, these animals compensate this lack with a highly diverse innate immune system against non-specific as well as specific targets [8]. Most of the biological and molecular biological studies on immune responses in sponges have been performed with the demosponge *Suberites domuncula*. For example, fungi are recognized by specific d-glucan carbohydrate receptors [9] and subsequently eliminated by expression of a fibrinogen-like protein. Gram-positive bacteria are killed by an increased induction of lysozyme [10], resulting in lysis of the microorganisms and subsequent phagocytosis. Gram-negative bacteria are recognized by sponges through a lipopolysaccharide-interacting protein and eliminated by expression of perforins through activation of a MyD88-dependent signaling pathway [11]. It should be noted that most of the recognition and effector molecules display high sequence similarities to polypeptides identified in deuterostomians. However, sponges also produce molecules that are only found in some protostomian taxa, e.g., a GlcNAc/GalNAc-binding lectin (tachylectin; [12]). It is amazing that this rich arsenal exists in the phylum Porifera which is the oldest, still extant, animal phylum, originating from the common metazoan ancestor, the Urmetazoa [13].

Considering the large and diverse number of noxious prokaryotic and eukaryotic organisms passing through sponges, it appeared to be compelling to search for antimicrobial peptides displaying broad activity spectra. It is well established that cationic antimicrobial peptides are produced by higher metazoans as host defense against microorganisms [14]. Recently they have been recognized as promising novel antimicrobial and antitumor agents [15]. The defensins and the ASABF (*Ascaris suum* antibacterial factor) are grouped to those peptides [16]. By screening the *S. domuncula* database that comprises over 40,000 sequences [17] several ESTs (Expressed Sequence Tag) were identified that displayed defensin-like sequence similarity. More specifically, the deduced protein shared highest relationship with the antimicrobial peptide designated as ASABF. ASABF peptides were first identified in *Ascaris suum* [18] and subsequently also in *Caenorhabditis elegans* [19]. The ASABF peptides and the defensins are both cysteine-rich. While the defensins are widespread both in protostomians and in deuterostomians [20], the ASABF peptides are restricted, among the triploblastic animals, to the nematodes [21]. In contrast to the defensins which comprise two to three antiparallel β-sheets that are linked together by three to four intramolecular disulfide bridges, the characteristic ASABFs have eight cysteine residues that form four disulfide bridges [22]. In the present study we report on the first sponge cDNA encoding an ASABF peptide. The predicted, mature peptide from *S. domuncula*, comprising 64 aa (amino acid) residues, was shown to be cationic (isoelectric point >8), a characteristic that it shares with the other known ASABFs [23]. Furthermore, we also checked the database from the Baikalian freshwater sponge *Lubomirskia baicalensis* for the existence of an ASABF-encoding cDNA; a cDNA fragment was found and completed. However, this sequence shared only little sequence similarity with other ASABFs or defensins.

The recombinant sponge ASABF protein was shown to be biologically active, as microbicidal (as expected) peptide possessing also hemolytic activity. Furthermore, we report here for the first time that an ASABF peptide is toxic also for metazoans. It is shown that the ASABF peptide was toxic for the marine gastropod/molluscs *Bittium* sp. which settled on apoptotic patches on the sponge surface [24]. The gastropod was attracted by the sponge after induction of the 3-hydroxyanthranilate 3,4-dioxygenase (*HAD*) gene, through quinolinic acid, a metabolite of the kynurenine pathway. In parallel with the increase in the steady-state level of *HAD,* the expression of a gene encoding a *caspase* was induced [25] that is known to be one key enzyme induced during apoptosis in *S. domuncula*. Both effector limbs, grazing of the gastropod and the caspase-dependent apoptosis pathway, led to the removal of the apoptotic tissue. Temporarily delayed, the expression of the *ASABF* gene encoding the functionally active peptide occurred, a process that resulted in the elimination of the gastropods. The data summarized show that in the sponge system the evolutionary most ancient ASABF peptide is involved not only in the microbicidal defense but also in the elimination of eukaryotic invaders. Both functions contribute to the rich arsenal of the innate immunity in the most basal metazoan phylum.

## 2. Results and Discussion

### 2.1. Sponge (*S. domuncula*, *L. baicalensis*) ASABF Peptide: Sequence Analysis and Phylogenetic Relationships

**Figure 1:**
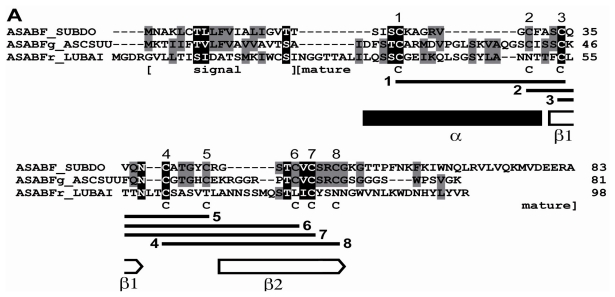

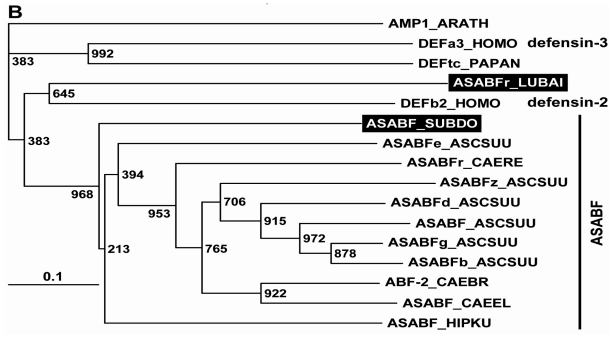
The *S. domuncula* ASABF peptide (ASABF_SUBDO), deduced from its cDNA (*SDASABF*). (**A**) The deduced protein was aligned with the most related sequence from the database, the ASABF-γ peptide from *A. suum* (ASABFg_ASCSUU; BAC00498.1) and the distantly similar sequence, the ASABF-related peptide from the Baikalian sponge *L. baicalensis* (ASABFr_LUBAI). Residues conserved (identical or similar with respect to their physico-chemical properties) in all sequences are shown in white on black; those which share similarity to at least two residues are in black on grey. The borders of the signal peptide and the mature peptide are given. The 8 Cys moieties that are involved in disulfide bridge formation are numbered; the connectivities of the Cys are indicated. The α-helix and the two β-strands are marked with a black solid bar and two open arrows, respectively; (**B**) These three proteins were compared with the related ASABFs from *A. suum*: ASABF-beta (ASABFb_ASCSUU; BAC00497.1), ASABF-delta (ASABFd_ASCSUU; BAC00499.1), ASABF-zeta (ASABFz_ASCSUU; BAC57992.1), ASABF-epsilon (ASABFe_ASCSUU; BAC41495.1) and the genuine ASABF (ASABF_ASCSUU; BAA11943.1); the ASABF from *C. remanei*: the CRE_25171/ASABF protein (ASABFr_CAERE; XP_003113198.1), the ASABF from *C. briggsae*: CBR-ABF-2 protein (ABF-2_CAEBR; XP_002646276.1), the ASABF from *C. elegans*: ASABF/abf-2 (ASABF_CAEEL; NP_491252.1), as well as the ASABF-like antimicrobial protein from *H. kuda* (ASABF_HIPKU; AAX58116.2). In addition, the two human defensins, beta defensin-2 (DEFb2_HOMO; AAC69554.1) and the neutrophil defensin-3 preproprotein (DEFa3_HOMO; NP_005208.1) as well as the theta defensin precursor from *P. anubis* (DEFtc_PAPAN; NP_001135410.1) were aligned. The plant putative defensin AMP1 protein from *A. thaliana* (AMP1_ARATH; AAM45086.1) was used as outgroup to root the tree. The scale bar indicates an evolutionary distance of 0.1 aa substitutions per position in the sequence.

By the technique of differential display of transcripts, in total RNA from a field specimen of *S. domuncula* 32 fragments were identified that were strongly upregulated, compared to the RNA from a specimen kept in the aquarium. After finding by this technique, the corresponding ESTs were also identified in the sponge database [17]. Then the cDNAs were completed. Blast similarity search of the deduced polypeptides revealed that two of those sequences shared highest sequence relationship with the *A. suum* ASABF-γ peptide from (BAC00498.1; [23]), and that the sequence from *S. domuncula* was complete; Figure 1A. Therefore, the sponge sequence was termed ASABF peptide (ASABF_SUBDO), deduced from the *SDASABF* cDNA. The similarity/identity score of the deduced peptide was 36%/27%. The open reading frame (ORF) of the 467 nt long cDNA ranges from nt_47–49_ to nt_296–293(stop)_ and comprises 83 aa contributing to a theoretical size of 9029 Da and an isoelectric point (pI) of 9.28. The peptide possesses one distinct signal sequence at the *N*-terminal end from aa_1_ to aa_19_ (SignalP 3.0 Server; Technical University of Denmark); Figure 1A. The length of this segment is identical to that predicted for the *A. suum* ASABF-γ peptide [23]; taking this sequence as reference, the predicted mature region starts at aa_20_ and ranges to the *C*-terminus. Hence, the theoretical M_w_ (molecular weight) is 7026, and the corresponding pI is 9.24. Therefore, the *S. domuncula* ASABF is a cationic protein, with a pI almost identical to that calculated for the mature ASABF from *A. suum* with 8.9 [23]. The *S. domuncula* ASABF sequence shows the characteristic consensus of the nematode CSαβ motif reading C-X(3,18)-C-X(3)-C-X(7,9)-[GS]-X-CX(4-13)-CXC [14]. Like the nematode peptide the mature *S. domuncula* ASABF sequence includes 8 Cys moieties (aa_23_, aa_30_, aa_34_, aa_39_, aa_44_, aa_49_, aa_51_, aa_54_) that allow the formation of disulfide bridges as highlighted in Figure 1A, and as predicted [21]. Secondary structure prediction was performed according to Cole *et al.* [26]. The characteristic CSαβ (cysteine-stabilized α-helix and β-sheet) structural motif [14] has been predicted according to Chou and Fasman [27]; Figure 1A. The *N*-terminal α-helix includes the first two Cys residues while the second β-strand comprises the last two Cys residues. No other domain could be identified in the sponge peptide [28].

The complete cDNA (*LBASABFr*), encoding the *L. baicalensis* ASABF-related peptide (ASABFr_LUBAI), has a length of 558 nts and an ORF spanning the segment nt_27–29_ to nt_321–323(stop)_, predicting a 98 aa moieties long peptide. The theoretical M_w_ is 10,665 and the pI was predicted with 7.61. Based on comparisons the signal peptide ranges from aa_1_ to aa_22_, followed by the mature peptide with an M_w_ of 8,172 and a theoretical pI of 7.86. This only slightly cationic predicted peptide has only 4 Cys residues.

In order to estimate the phylogenetic relationship of the sponge peptides to other metazoan sequences, for the alignment the highest related *A. suum*, *Caenorhabditis remanei*, *Caenorhabditis briggsae*, and the *C. elegans* sequences of the ASABF family, as well as the ASABF-related sequence from the seahorse *Hippocampus kuda* have been chosen [29]; additionally the two human defensins (the beta defensin-2 and the neutrophil alpha defensin-3 preproprotein) and the only metazoan theta defensin (from *Papio anubis*) were selected. The phylogenetic tree was established by using the plant ASABF-related putative defensin AMP1 protein from *Arabidopsis thaliana* as outgroup. The construction of the tree revealed that the ASABF sequences cluster together, including the sponge peptide (ASABF from *S. domuncula*) which forms the basis for that branch (Figure 1B). Separated from them are the human and baboon defensins. Interesting is the finding that the *L. baicalensis* ASABF-related peptide clusters together with the defensins that comprise likewise less Cys moieties in their mature peptide [23].

### 2.2. Preparation of Recombinant ASABF

**Figure 2:**
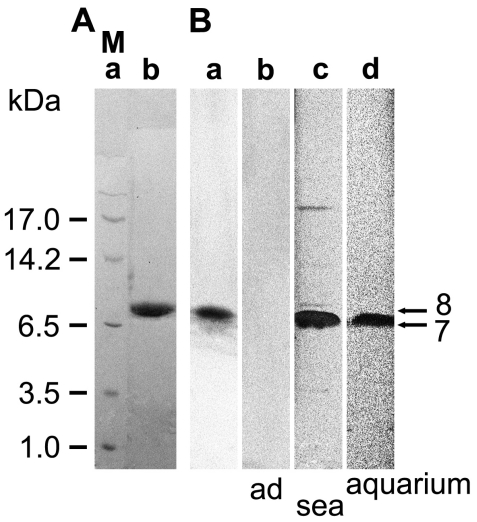
Analysis of recombinant and naturally occurring ASABF by SDS-PAGE. (**A**) *P. pastoris* cells were transformed with the *ASABF* cDNA (*SDASABF*), as described under “Experimental Section”. After purification, the recombinant protein was analyzed by SDS-PAGE. (**a**) Size markers (M); (**b**) Purified recombinant ASABF. After size separation the gel was stained by Coomassie brilliant blue; (**B**) (**a** and **b**) Western blot analysis of recombinant ASABF. The blot-transferred filters were incubated with (**a**) untreated PoAb-rASABFSUBDO, or (**b**) with PoAb-rASABFSUBDO adsorbed to rASABF_SUBDO; (**c** and **d**) Protein extracts from animals (**c**) sampled from the sea, or (**d**) kept for over 1 year in the aquarium were used for gel electrophoresis; 5 μg of protein were applied per slot.

Recombinant protein was prepared in the yeast *Pichia pastoris.* This eukaryotic expression system allows post-translational modifications such as disulfide bonding, which is required for proper folding of defensins. Accordingly, the complete ORF of *SDASABF*, without the putative signal sequence (*i.e.*, aa_20_ to aa_83_), was expressed as fusion to a 6xHis tag. Following Ni^2+^-NTA metal-affinity purification (Figure 2Ab), the recombinant protein (8.0 kDa (minus His tag: 7.1 kDa)) was used for immunization of rabbits. Then, specificity of the polyclonal anti-ASABF serum (PoAb-rASABFSUBDO) was assessed on Western blots (Figure 2Ba), showing a band at the expected size of the recombinant protein. In control experiments, 100 μL of the antibodies were adsorbed to 20 μg of the recombinant protein prior to their use. Consequently, no band could be observed on the immunoblots after application of these blocked antibodies (Figure 2Bb).

In a parallel set of experiments, the PoAb was applied to detect *S. domuncula* ASABF in protein extracts of sponge specimens that had been sampled from the sea (Figure 2Bc), or that had been cultured in aquaria for 12 months (Figure 2Bd). Thus, following SDS-PAGE and immunoblotting of identical protein concentrations, a band was detected with a size corresponding to the processed, mature variant of ASABF, lacking the signal sequence (*i.e.*, 7.0 kDa). The band intensity was considerably stronger in extracts of field specimens than in those of aquarium specimens.

### 2.3. Microbicidal Assay

The recombinant ASABF caused strong anti-microbial activity in the *in vitro* assay system (Table 1). Especially strong was the effect towards Gram-positive bacteria (*S. aureus*, *B. subtilis*, and *M. luteus*) with minimum inhibitory concentrations (MIC) between 2 and 5 μg/mL, while the activity against Gram-negative bacteria (*P. aeruginosa* and *E. coli*) was considerably lower with concentrations between 12 and 17 μg/mL. Interestingly, ASABF also caused distinct inhibitory effects on growth of eukaryotic fungi (*C. albicans* and *A. niger*) with MIC concentrations between 9 and 13 μg/mL. In order to prove that the effect measured was specific, the microorganisms were exposed to recombinant ASABF that had been adsorbed with antibodies prior to the addition to the cultures; such samples were found to be inhibitory only at MIC above 20 μg/mL.

### 2.4. Hemolytic Activity

**Figure 3:**
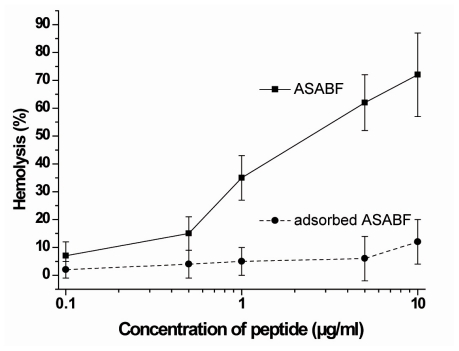
Hemolytic activity of ASABF, assayed in the human erythrocyte lysis assay. Red blood cells were incubated for 1 h (37 °C) at increasing concentrations of recombinant ASABF (—▪—). In a parallel series of experiments the recombinant ASABF was adsorbed to anti-ASABF antiserum (PoAb-rASABFSUBDO) (- - • - -). Subsequently, the lysed erythrocytes were determined on the basis of the released hemoglobin. The values represent the means ± SEM of ten experiments each.

As a convenient assay to study hemolysis human erythrocytes have been used and exposed to recombinant ASABF. The cells were exposed to the peptide and the percentage of the lysed erythrocytes was determined on the basis of the released hemoglobin. Under the assay conditions described under “Experimental Section” it was seen that the recombinant, non-adsorbed ASABF caused at a concentration of about 1 μg/mL 35% hemolysis. The degree of lysis increased to 76% at a concentration of 10 μg/mL (Figure 3). As a control, the ASABF peptide was adsorbed to anti-ASABF serum (PoAb-rASABFSUBDO), as described under “Experimental Section”. Using this ASABF sample the degree of lysis was very low and amounted to about 10% at the high concentration of 10 μg/mL.

### 2.5. Induction of Apoptosis in *S. domuncula* after Exposure of the Animals to Dip

**Figure 4:**
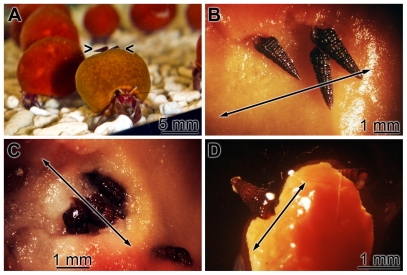
Colonialization of *S. domuncula* specimens by the gastropod *Bittium* sp. Specimens were kept in seawater in the presence of the chelator Dip. After 1 to 2 days in the aquarium the surfaces of the specimens developed speckled-type darker-colored areas which became apoptotic. (**A**) In an earlier stage one and soon after up to six gastropods colonized those apoptotic areas (> <); (**B** and **C**) Grazing gastropods on apoptotic areas (double-headed arrows); resulting in (**D**) a removal of those apoptotic areas (double-headed arrow).

If sponge specimens were kept in aquaria at lower aeration rate or in the presence of Fe^++^ chelator Dip the animals started to develop apoptotic spots/areas on their surface, as described [24]. Those regions were initially (after 1 day) marked by a deeper-red color. At a later stage the apoptotic surface regions became unstructured to meander-like. Previously, those regions had been identified to undergo apoptosis, as had been proven by the “Cell Death Detection ELISA test system” [24]. At day 2 these apoptotic regions were colonized by the gastropod *Bittium* sp. that had been attracted by quinolinic acid, a metabolite that is formed after induction of the 3-hydroxyanthranilate 3,4-dioxygenase (*HAD*) gene [24]. At days 2 to 3 the specimens of *Bittium* sp. (1 to 6 per 5 mm^2^) grazed on the apoptotic areas (Figure 4A); no gastropods were seen on non-apoptotic surfaces (not shown). As observed before, the gastropods ate (Figure 4B,C) and, by that, removed the apoptotic tissue (Figure 4D). After day 7 the previously apoptotic regions had been removed.

### 2.6. Expression of the *ASABF* Gene in *S. domuncula* Specimens: *in Situ* Hybridization

**Figure 5:**
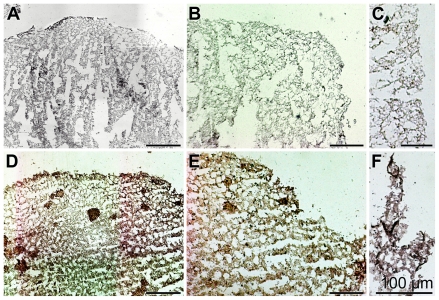
Expression studies of *ASABF* gene by application of the *in situ* hybridization technique. (**A**,**B**) Control sponge tissue of non-treated sponge specimens or (**C**–**F**) from animals kept for 1 day in the presence of the chelator Dip and subsequently for an additional 1 day in the absence of the chemical, was sliced and the sections were analyzed for the expression of the *ASABF* gene using the *SDASABF* DIG-labeled probe. In (**A**,**B**,**D–F**) hybridization was performed with the anti-sense ssDNA probe; (**C**) had been hybridized with the labeled sense probe. All size bars represent 100 μm.

Samples from a control specimen and from an animal kept for 1 day in Dip were analyzed by *in situ* hybridization. Using the DIG-labeled anti-sense ssDNA (*SDASABF*) probe the extent of reaction to the tissue structures was low in control sections (Figure 5A,B). In contrast, the reaction of the labeled probe with tissue slices from Dip-exposed animals was intense (Figure 5D,E); at a higher magnification the reaction with cell structures was evident (Figure 5F). One control was performed by hybridizing a sample from the Dip-treated animal with a labeled sense ssDNA probe; there, only background signals were recorded (Figure 5C).

### 2.7. Expressions of the *HAD*, the *ASABF* as well as the *Caspase* Genes: Determination by qRT-PCR

**Figure 6:**
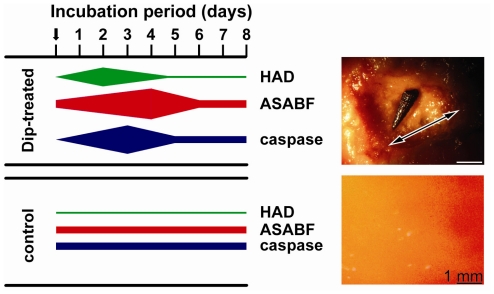
Diagrammatic sketch of the expression levels of *HAD*, *ASABF* as well as of the *caspase* gene in Dip-treated and in control specimens (left). The widths of the bars symbolize the level of expression. At the upper right an image of the surface of a 3-day-old apoptotic area (double-headed arrow) with a gastropod is shown; lower right shows the plain surface of a non-treated specimen.

Tissue samples from apoptotic areas on the surface of Dip-treated animals, as well as from control animals were taken and subjected to qRT-PCR analysis. For a quantitative assessment steady-state gene expressions of *HAD* (marker for quinolinic acid synthesis), the *caspase* (formation of apoptotic tissue [30]) as well as of *ASABF* (ASABF synthesis) were chosen. The tissue samples for the determination of the respective transcript levels in apoptotic areas were taken from the rim region of those regions. The determinations were performed at the beginning of the exposure (day 0), as well as after 1 to 9 days. The results showed that the expression level of *HAD* at the beginning of the experiments was approximately 2.1 × 10^−3^ with respect to the expression of the house-keeping gene *tubulin* (Table 2). While in the controls the expression level did not significantly change during the 9-day incubation period, a strong increase in the steady-state expression of *HAD* was measured in the Dip-treated animals. The maximum of expression was seen already at day 2 (27.1 × 10^−3^); during the end of the experiment (after 9 days) the expression level returned to normal (3.1 × 10^−3^). Focusing on *ASABF* expression, likewise no change of the steady-state expression was observed in the controls (around 1.3 × 10^−2^). In the Dip-treated specimens a strong and significant increase was found already after day 1 (2.8 × 10^−2^), while the maximum was seen 4 days after exposure to Dip (9.8 × 10^−2^). Subsequently, the level of expression decreased. Finally, the expression of the *caspase* gene likewise increased in the Dip-treated animals from 1.5 × 10^−4^ (day 0) to 12.4 × 10^−4^ at day 3 (Table 2). In the control tissue no significant changes were measured. A diagrammatic sketch, summarizing the expression levels in apoptotic tissue from the surface of Dip-treated specimens, as well as from controls, is given in Figure 6. There, also the surface texture of a 3-day-old apoptotic area with a grazing gastropod is shown with its uneven appearance in contrast to the smooth/plain surface in the control.

**Figure 7:**
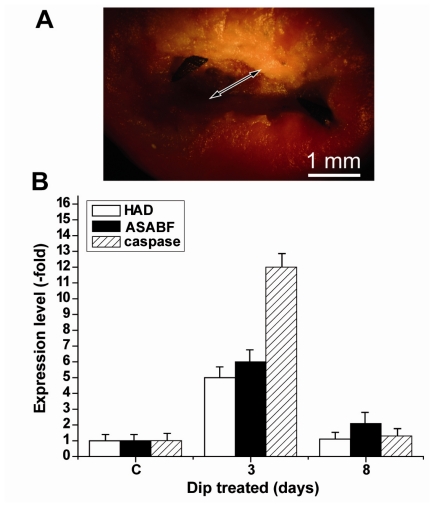
Spatially resolved analysis of the expression level of *HAD*, *ASABF* as well as of the *caspase* gene. Tissue samples from controls as well as samples from within the apoptotic areas (the diameter of the area is given by a double-headed arrow) of Dip-treated specimens were analyzed. The expression is given in arbitrary units as multiple-fold of the transcript levels seen in the controls (non-treated animals; day 0). The analyses were performed by qRT-PCR 3 days and 8 days after exposure of the specimens to Dip using the expression of the *tubulin* gene as a reference house-keeping gene; the controls remained untreated. The means ± SEM (*n* = 5) are given.

For the expression studies in the above described series of experiments tissue from the rim regions had been selected. To obtain a more detailed view of the expression levels of *HAD*, *ASABF* and *caspase* tissue samples were taken from within the apoptotic area. Such a spatially resolved study was only possible after a more progressed stage of apoptosis, 3 days after Dip exposure. In comparison, tissue samples from apoptotic regions after their ablation through the gastropods were analyzed (Figure 7). The data revealed that at day 3 the increase in the steady-state level of expression of the *HAD* gene and the *ASABF* gene was 4.8-fold and 5.9-fold, respectively, while the increase of the expression level for the *caspase* gene was much higher with 12.2-fold.

### 2.8. Acute/Subchronic Toxicity Testing of the Gastropod

The above outlined results indicate that the drop of the *HAD* expression coincided (after about 4 days after exposure to Dip) with the maximum of the expression of the *ASABF* gene, encoding the antimicrobial peptide (Figure 6). In addition, it is seen that after about 5 days the gastropods disappeared from the previously apoptotic areas. The reason for the elimination of the gastropods is not quinolinic acid, since this dicarboxylic acid is not toxic for those epibionts. Therefore, it was intriguing to check if it was the ASABF peptide which may have caused a toxic effect. Accordingly it was necessary to test if the recombinant peptide affected the viability of the gastropods.

**Figure 8:**
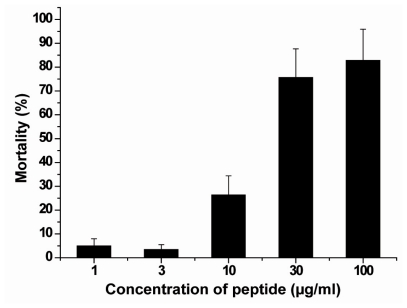
Acute/subchronic toxicity of recombinant ASABF peptide on the gastropod *Bittium* sp. The gastropods were incubated for 4 days with the recombinant peptide. Subsequently cultivation of the animals continued for 4 days in seawater without the peptide; the mortality was determined as described under “Experimental Section”.

As outlined in the assay conditions described under “Experimental Section”, the gastropods were exposed for 96 h to ASABF in a serial concentration range. After incubation the mortality in the assays with ASABF were determined to be; at 3 μg/mL: 3.5 ± 2%, 10 μg/mL: 26.4 ± 8% and 30 μg/mL: 75.7 ± 12%. In the controls (untreated animals) 100% of the animals survived. According to the standards the toxicity of the ASABF towards the gastropods should be assessed as “highly toxic” (US EPA 2011); Figure 8.

## 3. Discussion

Antimicrobial agents that usually represent peptides with a length of less than 50 amino acid residues are potent effector molecules of the innate immune system. They interact with both microbial and eukaryotic cell membranes and, in turn, cause cell lysis. Besides direct interactions with biomembranes, they function in vertebrates also as mediators of inflammation, cause cytokine release, affect cell proliferation, and interfere with angiogenesis, wound healing, or chemotaxis [31,32]. Most of those peptides are classified to the group of cationic peptides [33], comprising characteristic domains, e.g., the cysteine-rich CSαβ-type motif [14]. Already 20 years ago it had been proposed that those “antimicrobial peptides” act not only on cell membranes but also affect intracellular targets, as has been shown e.g., for the small tachyplesin peptides from horseshoe crabs [34]. The remarkable feature of those antimicrobial peptides is their property to act in a broad spectrum against Gram-positive and Gram-negative bacteria as well as fungi [35].

Even though it is known that sponges are rich sources of bioactive compounds of low molecular weight [3], no screening for genes encoding antimicrobial peptides from the sponges themselves has yet been reported. Unlike the antibiotic peptides, synthesized by the sponge symbionts and encoded by them, e.g., by synthases [36], the amino acid sequences of the antimicrobial peptides are encoded by the host organism, the sponge itself. Hence, such antimicrobial peptides can be considered to be components of the metazoan innate or natural immune system which protects them against prokaryotic and eukaryotic invading organisms. The present contribution reports on a gene from *S. domuncula*, encoding an ASABF-type antimicrobial peptide that acts against microorganisms as well as against eukaryotic cells. The *S. domuncula* ASABF is a cationic peptide that comprises eight cysteines allowing the formation of four intramolecular disulfide bridges. From the ORF of the full-length cDNA sequence an 83-residue precursor peptide can be predicted. From this polypeptide a signal peptide of 19 amino acids in length is cleaved off, converting the precursor into a 64 aa long, mature peptide. The four disulfide bridges follow the prediction based on the nematode ASABFs [21]. The sequence also shares the CSαβ motif with the nematode ASABFs. The sponge polypeptide is, like the other ASABFs, related to the defensins which comprise 3–4 intramolecular disulfide bridges [22]. The phylogenetic analysis likewise indicated a separation of the ASABFs, including the sponge sequence, from the defensins, regardless of whether they come from vertebrates or invertebrates. Like the ASABFs the defensins are key elements of the immune system and provide a broad-range defense against eukaryotic and protozoan pathogens [16]. It is important to mention that in metazoans the defensins are expressed cell-type/tissue specifically and often show modulatory effects on immune cells, e.g., macrophages, lymphocytes or mast cells [37]. Even more, an interference of the defensins with host cell recognition proteins has been described [38]. These findings on the sequence and function polymorphism of the ASABFs/defensins underscore their important role in the innate immune system of metazoans.

The sponge ASABF has been successfully expressed in the *P. pastoris* system and the biologically active peptide was obtained. The recombinant peptide turned out to be highly active against Gram-positive bacteria with a MIC concentration between 2 and 5 μg/mL (0.3 to 0.7 μM), and is, hence, similarly active as the antimicrobial nematode ASABF peptides, abf-1 and abf-2 [19]. Less effective is the sponge peptide against Gram-negative bacteria with >12 μg/mL. In addition, microbicidal activity was seen in eukaryotic fungal systems (*C. albicans* and *A. niger*) with an inhibitory activity at 9–13 μg/mL. Considering the screening range of Zhang *et al.* [39] we also tested the sponge peptide in the human hemolysis system with erythrocytes. Interestingly, even at the low concentration of 1 μg/mL (0.15 μM) the sponge peptide caused a significant hemolytic activity; about 30–40% of the erythrocytes were found to be lysed at that concentration. This efficiency is considerably higher than that known for the nematode *A. suum* ASABFs [39], which varies around a MIC of 10–30 μg/mL.

The potent hemolytic activity prompted us to determine the effect of the recombinant sponge ASABF on the gastropod *Bittium* sp. an eukaryotic epibiont that occasionally grazes on *S. domuncula*. Consequently we had to answer two questions. First, the determination of the gene expression status of *ASABF* in a control region and in the areas where the gastropod settled, and second, of the potential toxic effect of the recombinant ASABF peptide on the gastropod directly. Previously it had been shown that this gastropod settles on the surface of *S. domuncula* after induction of apoptosis in response to a lower oxygen partial pressure [24]. In this previous report apoptosis was measured with the “Cell Death Detection ELISA test system” [40] that bases on the quantification of the degree of chromatin fragmentation to mono- and oligonucleosomes. During the process of apoptosis the expression of the gene for the key enzyme of the quinolinic acid pathway is induced, the 3-hydroxyanthranilate 3,4-dioxygenase (*HAD*) that converts 3-hydroxyanthranilate to 2-amino-3-carboxymuconate 6-semialdehyde under consumption of oxygen. Since the latter metabolite is not stable, it non-enzymatically converts to quinolinic acid [41]. This dicarboxylic acid is not only in sponges [24] but also in human cells a selective inducer of apoptosis [42]. In sponges the initiating enzyme, HAD, is rapidly induced during 2 days after impairing the environment of the animals. After formation of quinolinic acid in the apoptotic tissue, the gastropod *Bittium* sp. colonizes that region, very likely attracted by the dicarboxylic acid. Subsequently, as implicated [24], the gastropods ablate the apoptotic tissue during a period of 2–3 days. In parallel with the induction of *HAD*, a significant upregulation of the steady-state expression of *caspase* occurs. Increased transcript levels of *caspase* in apoptotic tissue are also known from earlier studies [30]. After a lag period of 2 days after the induction of *HAD*, the gene encoding the ASABF peptide is induced in the tissue, surrounding the apoptotic area. The expression of *ASABF* in apoptotic tissue is low, almost not existing, while it is reasonably expressed in control, non-apoptotic tissue (data not shown). However, if samples are taken from regions bordering the apoptotic area that has been eliminated by the gastropods, a strong upregulation is seen. The induction of the gene for the ASABF peptide has been described previously in the nematode *A. suum*, after exposure of the worm to heat-killed bacteria [23].

The toxicity of the ASABF peptide on the gastropod *Bittium* sp. has been determined under controlled, closed conditions. Our toxicity studies revealed that ASABF is already lethal for the animals at a concentration of the recombinant peptide as low as 10–30 μg/mL during an incubation period of 4 days. This first description of an antimicrobial peptide from sponges which is toxic for animal cells and is likely used as a defense against attacking predators, widens the view of the activity spectra of the “antimicrobial” peptides. Consequently, the sponge ASABF, a member of the innate immune system, most likely acts as a broad-spectrum defense molecule. The perhaps ubiquitous presence of antimicrobial peptides of the ASABF type in sponges is supported by the identification of the ASABF-related gene in the freshwater sponge *L. baicalensis*.

## 4. Experimental Section

### 4.1. Chemicals, Materials and Enzymes

The sources of the materials used for this study were given previously [43]. In addition, Dip (2,2′-dipyridyl) was purchased from Sigma/Aldrich (St. Louis, MO; USA), and PCR DIG (digoxigenin) Probe Synthesis Kit, anti-DIG AP Fab fragments, as well as disodium 2-chloro-5-(4-methoxyspiro {1,2-dioxetane-3,2′-(5′-chloro)tricyclo[3.3.1.1^3,7^]decan}-4-yl)-1-phenyl phosphate (CDP-Star) were obtained from Roche (Mannheim; Germany).

### 4.2. Sponges and Gastropod/Mollusc Colonization

Specimens of the marine sponge *S. domuncula* (Porifera, Demospongiae, Hadromerida) were sampled in the Northern Adriatic near Rovinj (Croatia), and then kept in aquaria in Mainz (Germany) at a temperature of 17 °C for more than 12 months. Extracts were prepared from tissue samples by treatment with four parts of a 0.4 M Tris-HCl buffer (pH 8.0; supplemented with 50 mM NaCl, 0.5 mM EDTA and 5 mM MgCl_2_). After grinding, and stirring of the suspension at 2 °C for 1 h a clear supernatant was obtained after centrifugation (20,000 × *g*, 30 min, 2 °C); this was collected and contained between 3 and 7 mg of protein per mL. This extract was diluted down to 2 mg/mL and used for the experiments.

The experiments were performed in 5 L (artificial sea water [24]) aquaria, containing 3 sponges each. Apoptosis was induced in sponges by keeping the specimens in the presence of 50 μg/mL of the Fe^++^ chelator Dip [44] for 1 day. Under these conditions the specimens responded with the formation of speckled patches on their surfaces, as described earlier [24]. By application of the “Cell Death Detection ELISA test system” (Roche) apoptosis was proven in these regions [24]. To each aquarium, both controls and Dip (2,2′-dipyridyl) supplemented ones, approximately 100 specimens of the gastropod/mollusc *Bittium* sp. (Mollusca, Gastropoda, Cerithiidae, Bittium) were added. Already 1 day later the apoptotic patches on the sponge surfaces were covered by 1 to 6 specimens of *Bittium* sp. per 5 mm^2^ (Dip-treated), while on the surfaces of specimens kept in absence of Dip (controls) no gastropods were seen. As described before, the gastropods ablated the apoptotic tissue within 3 days. Seven days after the beginning of the experiments no signs of apoptotic tissue could be seen. Tissue samples were taken at the beginning of the experiments as well as after 1 day, 2 days, 3 days, 4 days, 5 days, 7/8 days, and 9 days from Dip-treated (speckled-type regions) and from control regions, and analyzed for gene expression.

### 4.3. Maintenance of the Gastropod *Bittium* sp

*Bittium* sp. (Gastropoda: Cerithiidae) are common grazers, epizoons [45], abundantly seen on the surfaces of *S. domuncula* apoptotic specimens. The gastropods were collected, transferred to nets (mesh size 44 μm) and could be kept in aquaria, for over 30 days, without losing more than 10% of the specimens. Routinely, the gastropods were used for the experiments 6 days after collection from the apoptotic specimens.

### 4.4. Identification and Cloning of the Poriferan *ASABF* Peptides

*S. domuncula* ASABF: For the identification of the cDNAs encoding putative defensin-like molecules the technique of differential display was employed according to Müller *et al.* [4]. Total RNA was extracted from *S. domuncula* specimens that had been sampled from the sea or had been cultivated in aquaria for more than one year. Specimens that had been taken from nature showed a higher load of bacteria and, in turn, were more likely to express those polypeptides in a wider array of antibacterial defense molecules and at higher expression levels than aquarium animals [46]. The cloning procedure, given in details before [4] is outlined in brief: 1.8 μg of total RNA of aquarium animals or of field specimens was reverse transcribed using the anchored oligo(dT) primer T_11_CC and SuperScript III Reverse Transcriptase (Invitrogen, Darmstadt; Germany). Following first-strand cDNA synthesis, the samples were diluted 1:10 with H_2_O and, then, subjected to polymerase chain reaction (PCR). For this reaction, Platinum Taq DNA polymerase (Invitrogen) was employed in combination with the IR-labeled arbitrary primer 5′-GTGATCGCAG-3′ and an oligo(dT) primer. After initial denaturation at 94 °C for 2 min, cDNA was amplified during 38 cycles each, at 94 °C for 30 s, 42 °C for 120 s, and 72 °C for 30 s, followed by a final incubation of 10 min at 72 °C. Subsequently, amplicons were size-separated during polyacrylamide gel electrophoresis, using an automated DNA sequencer (LI-COR 4300 DNA Analysis System). Then, cDNAs between 250 and 400 nucleotides (nt) of differentially expressed transcripts were excised using the Odyssey (LI-COR) infrared imaging system and subsequently reamplified by PCR. Finally, the products were sub-cloned in the pGEM-T vector (Promega) and sequenced. Complete cDNAs were obtained by primer walking [47]. Thus, the clone *SDASABF* was identified (among 32 differentially expressed transcripts found within field animals), comprising 467 nts (excluding the poly(A) tail) with an ORF encoding the putative protein ASABF_SUBDO.

*L. baicalensis* ASABF: Following the identification of *S. domuncula* ASABF, >2500 *L. baicalensis* ESTs [28] were screened for related sequences. One clone was discovered, comprising 558 nts with considerable sequence homology to *SDASABF*, which was termed *LBASABFr*. The ORF was found to be complete.

### 4.5. Sequence Analyses

Homology searches were conducted through servers at the European Bioinformatics Institute, Hinxton, United Kingdom and the National Center for Biotechnology Information (NCBI), Bethesda, MD [29]. Multiple alignments were carried out with ClustalW version 1.6 [48]. Phylogenetic trees were constructed on the basis of aa sequence alignments applying the Neighbor-Joining method to distance matrices that were calculated using the Dayhoff PAM matrix model [49,50]. The degree of support for internal branches was further assessed by bootstrapping [51]. The graphical output of the bootstrap figures was produced through the “Treeview” software [52] and GeneDoc [53]. Potential domains, subunits, patterns, and transmembrane regions were predicted searching the Pfam [54] or the SMART database [55].

### 4.6. Preparation of Recombinant *S. domuncula ASABF* Peptide

cDNA of *S. domuncula* ASABF, coding for the complete mature protein (aa_20_ to aa_83_), was cloned as gene of interest into the *pPIC3.5* yeast expression vector (Invitrogen) via *Sna*B I and *Eco*R I. At the 5′ terminus, an ATG initiation codon was placed within the context of a yeast translation initiation sequence ([56] and according to the manufacturer’s protocol). Additionally, the cDNA contained a 3′ 6xhistidine (His) tag coding sequence (to facilitate metal-affinity chromatography purification of the recombinant protein), followed by a stop codon. After digestion with *Sal* I, chemically competent *Pichia pastoris* GS115 cells were transformed with the linearized construct. Subsequently, transformants were selected for their ability to grow on histidine-deficient medium. For recombinant protein expression, transformants were cultured for 84–96 h at 30 °C in appropriate medium with 0.5% (v/v) methanol. Then, cells were harvested, transferred into Breaking Buffer (50 mM sodium phosphate pH 7.4, 1 mM PMSF (phenylmethylsulfonyl fluoride), 1 mM EDTA, 5% glycerol), and homogenized (Precellys; PeqLab, Erlangen; Germany). Finally, the recombinant protein (termed rASABF_SUBDO) was purified through nickel-nitrilotriacetic acid (Ni^2+^-NTA) metal-affinity chromatography and checked through SDS-PAGE.

### 4.7. Preparation of Antibodies

The purified, recombinant ASABF protein (rASABF_SUBDO) was used for the production of polyclonal antibodies (PoAb), as described [8,57]. Thus, female rabbits (White New Zealand) were immunized thrice with 20 μg/boost. Following collection of the PoAb (termed PoAb-rASABFSUBDO) the titer was determined to be 1:5,000. For control experiments, 100 μL of PoAb were incubated with 20 μg of the antigen during an incubation period of 1 h (4 °C). Only then (adsorbed antibody preparation) these blocked antibodies were applied to immunodetection assays.

### 4.8. SDS-PAGE and Immunodetection Analysis

Sodium dodecyl sulfate polyacrylamide gel electrophoresis (SDS-PAGE) was routinely performed as follows. Samples of 6 μg of protein were mixed with loading buffer (Roti-Load; Carl Roth, Karlsruhe; Germany), boiled for 8 min, and subjected to SDS-PAGE (15% acrylamide and 0.1% SDS) as described [58]. The gels were stained with Coomassie brilliant blue. The protein size standard “Dual Color” (Carl Roth) was used to estimate protein sizes. For Western blot analyses, size-separated proteins were transferred to PVDF-Immobilon membranes. Then, the membranes were blocked at room temperature with blocking solution (Roche; 1% (v/v) in TBS-T buffer; 20 mM Tris-HCl pH 7.6, 137 mM NaCl, 0.1% (v/v) Tween-20). Following consecutive incubations with PoAb-rASABFSUBDO (1:3,000 dilution in TBS-T, supplemented with 0.1% (v/v) Blocking solution), alkaline phosphatase (AP)-conjugated species-specific secondary antibodies (1:4000 dilution), and 4-nitro blue tetrazolium chloride (NBT)/5-bromo-4-chloro-3-indolyl phosphate (BCIP) (Invitrogen), proteins were detected colorimetrically. The “Ultra-Low Range Marker” (Sigma; M3546) was used to determine the apparent size of the peptide.

### 4.9. Quantitative Real-Time RT-PCR (qRT-PCR)

Quantitative real-time RT-PCR (qRT-PCR) was applied to determine the steady-state expression of the gene encoding ASABF (*SDASABF*), as recently described in detail [59,60]. Following extraction of tissue RNA was treated with DNAse to eliminate contaminating genomic DNA. First strand cDNA synthesis was performed by SuperScript III Reverse Transcriptase in a reaction mixture containing 5 μg of total RNA, dNTPs, oligo(dT)_18_, and reverse transcriptase buffer (Invitrogen) at 42 °C for 1 h. After enzyme inactivation (65 °C, 15 min), qPCR experiments were performed in an iCycler (Bio-Rad), using 1/10 serial dilutions in triplicate as described [61]. After appropriate dilution, 2 μL of the reaction mixture were employed as a template for 30 μL qPCR assays. Each reaction contained “Absolute Blue SYBR Green” master mixture (ABgene, Hamburg; Germany) and 5 pmol of each primer. All reactions were run with an initial denaturation at 95 °C for 3 min, followed by 40 cycles each, of 95 °C for 15 s, 62 °C for 30 s, 72 °C for 35 s, and 80 °C for 20 s. The following primers were used for amplification of *SDASABF*: Fwd: 5′-GCAAAGCTGGCAGAGTTGGAT-3′ (nt_114_ to nt_134_) and Rev: 5′-AGATTTTGAACTTGTTGAAAGG-3′ (nt_245_ to nt_224_); the size of the fragment obtained was 132 bp. For the amplification of the *S. domuncula* caspase gene *SDCASPL* (NCBI accession number AM392430.1; [8]) Fwd: 5′-GGACAAACAGATGTACACAGAG-3′ (nt_597_ to nt_618_) and Rev: 5′-GATGTAGTTTCCTTCTGGAGGT-3′ (nt_732_ to nt_711_) were applied, resulting in a fragment of 136 bp. Finally, the expression level of the *S. domuncula* 3-hydroxyanthranilate 3,4-dioxygenase (*HAD*; AJ298053.1) was quantified using the primer pair Fwd: 5′-CTCAGGTACTATGTTGATGGTA-3′ (nt_369_ to nt_390_) and Rev: 5′-TGGACTTCCATTCTTGTGTTCC-3′ (nt_300_ to nt_279_); size of the fragment, 132 bp. The expression of the house-keeping gene *β-tubulin* (AJ550806; [62]) was determined with the following primers, Fwd: 5′-AACCGCTGTTTGCGACATCC-3′ (nt_1,126_ to nt_1,145_) and Rev: 5′-CAATGCAAGAAAGCCTTTCGCC-3′ (nt_1266_ to nt_1245_); fragment size, 141 bp. The threshold position was set to 50.0 RFU above PCR subtracted baseline for all runs. Mean Ct values and efficiencies were calculated by the iCycler software. Expression levels of the respective transcripts were correlated with the one for *β-tubulin* to assess the relative expression levels (E_Tub_ ^CtTub^/E_ASABF_ ^CtASABF^; E_Tub_ ^CtTub^/E_Casp_ ^CtCasp^; E_Tub_ ^CtTub^/E_HAD_ ^CtHAD^); whereby “E” describes the PCR efficiency and “Ct” represents the threshold cycle.

### 4.10. *In Situ* Hybridization

*In situ* hybridization assays of *S. domuncula* tissue slices were performed according to Schröder *et al.* [62]. Briefly, labeling of ssDNA probes (*SDASABF*) was carried out with the PCR DIG Probe Synthesis Kit (Roche), using either forward primer 5′-ATGAATGCAAAGTTGTGCACTC-3′ (nt_47_ to nt_68_; for the sense probe) or reverse primer 5′-CAAGCGCGTTCTTCATCGAC-3′ (nt_297_ to nt_278_; for the anti-sense probe) in combination with a linearized template. The resulting labeled ssDNA probes spanned nt_47_ to nt_297_ of the *SDASABF* cDNA. Then, cryosections (8 μm thickness) were fixed with paraformaldehyde, washed with PBS (10 mM sodium phosphate pH 7.4, in 150 mM NaCl) at room temperature, and incubated with Proteinase K. For rehydration, the sections were hybridized with the anti-sense or sense probe (negative control) overnight at 40°C. After blocking, the sections were incubated for 1 h at 37 °C with anti-DIG Fab fragments (conjugated to alkaline phosphatase) and, then, washed twice. For visualization of the signals, the dye reagents BCIP/NBT (5-bromo-4-chloro-3′-indolyphosphate/nitro-blue tetrazolium) were used in a Tris-buffer (100 mM Tris-HCl pH 9.5, 100 mM NaCl, and 50 mM MgCl_2_).

### 4.11. Microbicidal Assay

Microbicidal/antibacterial assay was performed as earlier described [63,64] using the Mueller Hinton Medium (DIFCO Invitrogen, Karlsruhe; Germany) at pH 7.0. The following microorganisms were tested: the Gram-positive bacteria *Staphylococcus aureus* ATCC 25293, *Bacillus subtilis* ATCC 6633, and *Micrococcus luteus* ATCC 9341, the Gram-negative bacteria *Pseudomonas aeruginosa* ATCC 9027, and *Escherichia coli* ATCC 25922), and the fungi *Candida albicans* ATCC 10231 and *Aspergillus niger* ATCC 16404. The minimal inhibitory concentration (MIC) was determined and the values were given in μg/mL.

### 4.12. Hemolytic Assay

Human type A erythrocytes were used for the hemolytic assay; they came from healthy individuals. The detailed assay has been described earlier [39,65]. PBS-washed erythrocytes were used to prepare a 10% (v/v) suspension by using an isotonic glucose phosphate buffer (1 mM K-phosphate buffer, pH 7.0; supplemented with 287 mM glucose). Serial dilutions of recombinant non-adsorbed or adsorbed ASABF were prepared to a final volume of 200 μL in 96-well V-bottomed microtiter plates (Nunc/Thermo Electron, Langenselbold; Germany). To reach total hemolysis the samples were subjected to 1% (v/v) Triton X-100. After incubation for 1 h (37 °C) the plates were centrifuged (10 min at 2000 × *g* at 20 °C) and a 150 μL aliquot from the supernatant was taken and used for absorbance measurements at 450 nm. The percentage of hemolysis was calculated as described [65] $((A_{450}\;\text{of the ASABF}-\text{treated sample}-A_{450}\;\text{of buffer}-\text{treated sample})/(A_{450}\;\text{of Triton X}-100-\text{treated}\;\text{sample}-A_{450}\;\text{of buffer}-\text{treated sample}))\times 100\%$.

### 4.13. Acute/Subchronic Toxicity Testing

The gastropods, collected from one affected specimen of *S. domuncula*, were exposed at 17°C to 3 μg/mL, 10 μg/mL, or 30 μg/mL of recombinant ASABF in seawater, or remained without this peptide. The animals, 10 specimens per assay, were kept in parallel in aerated aquaria (volume of 200 mL) for 96 h. Subsequently the gastropods were transferred to seawater without the peptide and culture continued for 96 more hours. Then the living gastropods were counted. The means ± standard deviations of three series of experiments were calculated by using the paired Student’s *t*-test [66].

### 4.14. Further Methods

For the quantification of protein, the Bradford method ([67]; Roti-Quant solution-Roth) was used.

## 5. Conclusion

**Figure 9:**
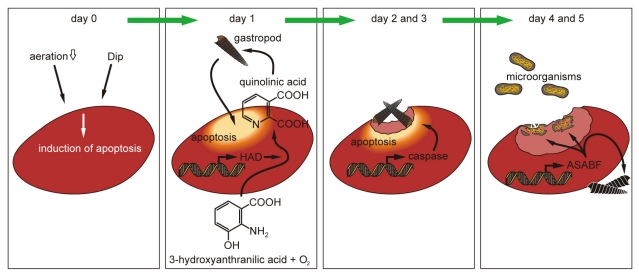
Schematic outline of the elimination of apoptotic tissue from the sponge *S. domuncula*, after induction with the Fe^++^ chelator Dip. Initially the expression of the *HAD* gene is induced as a response to Dip. After initiation of Dip-induced apoptosis the *caspase* gene is upregulated and, sequentially, the expression of the *ASABF* gene sets on. While quinolinic acid, a metabolite synthesized via the HAD/kynurenine pathway, attracts the gastropod *Bittium* sp. the terminally expressed ASABF causes, after removal of the apoptotic tissue, an repelling of the gastropods as well as a killing of the microorganisms.

In the present study we describe, for the first time, a sponge ASABF peptide and show that the polypeptide, deduced from the complete cDNA, comprises the characteristic features of ASABF peptides so far identified especially in nematodes. The induction of the *ASABF* gene was monitored in tissue undergoing apoptosis. In those apoptotic regions the expression of *ASABF* is preceded by the expression of *HAD*, which encodes a key enzyme involved in the formation of quinolinic acid, and in parallel by the expression of the *caspase* gene that initiates and regulates the onset and the completion of the apoptotic process. The elimination of the apoptotic tissue is supported by the gastropod *Bittium* sp. which is attracted by the initially synthesized quinolinic acid and finally eliminated by ASABF. In parallel to the elimination of the gastropod, also potentially attacking microorganisms are killed by ASABF. Our data demonstrate that the innate immune network, based on antimicrobial peptides, in sponges is complex and together with the host-encoded immune defense arm, ASABF, involves a transient grazing of symbiotic gastropods that accelerate the elimination of apoptotic tissue (Figure 9).

## Figures and Tables

**Table 1:** Antimicrobial activity of non-adsorbed or antibody-adsorbed recombinant ASABF against Gram-positive bacteria, Gram-negative bacteria and two fungi. The inhibition is given as minimum inhibitory concentrations (MIC) value in μg/mL.

Microorganism		Antimicrobial activity MIC (μg/mL)	

		Non-Adsorbed ASABF	Adsorbed ASABF
Gram-positive bacteria:	*Staphylococcus aureus*	1.7 ± 0.5	>20
	*Bacillus subtilis*	4.8 ± 0.6	>20
	*Micrococcus luteus*	3.6 ± 1.0	>20
Gram-negative bacteria:	*Pseudomonas aeruginosa*	12.4 ± 5.5	>20
	*Escherichia coli*	17.4 ± 6.0	>20
Fungi:	*Candida albicans*	8.5 ± 4.0	>20
	*Aspergillus niger*	12.9 ± 4.0	>20

**Table 2:** Expression levels of *S. domuncula HAD*, *ASABF* as well as of the *caspase* genes on the surfaces of control animals, not treated with the Fe^++^ chelator, or treated with Dip as described under “Experimental Section”. Tissue samples from the Dip-treated animals were taken from the rim of apoptotic region, or after day 5, from the area which previously had been apoptotic and was then cleaned by the gastropod. Then, RNA was extracted and expression levels were quantified through qRT-PCR. Each data point represents the mRNA level of the respective expressed gene (x: *HAD*, *ASABF* or *caspase*) normalized to the amount of *β-tubulin* transcripts, as means ± SEM (*n* = five experiments per time point).

Time point (days)	Gene expression: (mRNA_x_ ± SEM)/mRNA*_tubulin_*					

	*HAD*		*ASABF*		*Caspase*	

	Control (×10^−3^)	Dip-Treated (×10^−3^)	Control (×10^−2^)	Dip-Treated (×10^−2^)	Control (×10^−4^)	Dip-Treated (×10^−4^)
0	2.1 ± 0.3	2.2 ± 0.3	1.3 ± 0.2	1.3 ± 0.2	1.3 ± 0.1	1.5 ± 0.2
1	2.3 ± 0.3	5.3 ± 0.6	0.9 ± 0.1	2.8 ± 0.3	1.6 ± 0.2	6.7 ± 0.7
2	2.4 ± 0.4	27.1 ± 3.8	1.2 ± 0.2	4.0 ± 0.5	1.9 ± 0.2	9.3 ± 1.0
3	1.9 ± 0.2	19.9 ± 1.7	1.7 ± 0.2	6.3 ± 0.8	1.8 ± 0.1	12.4 ± 1.7
4	1.9 ± 0.2	7.2 ± 0.9	1.6 ± 0.2	9.8 ± 0.9	1.4 ± 0.3	6.9 ± 0.8
5	2.2 ± 0.5	4.2 ± 0.6	2.1 ± 0.3	6.9 ± 0.7	1.8 ± 0.2	1.7 ± 0.1
7	2.3 ± 0.3	3.4 ± 0.4	1.9 ± 0.4	3.7 ± 0.5	1.8 ± 0.2	1.5 ± 0.2
9	2.5 ± 0.4	3.1 ± 0.3	1.8 ± 0.2	4.4 ± 0.5	2.1 ± 0.3	1.9 ± 0.3
